# *ERO1L* Is a Novel and Potential Biomarker in Lung Adenocarcinoma and Shapes the Immune-Suppressive Tumor Microenvironment

**DOI:** 10.3389/fimmu.2021.677169

**Published:** 2021-07-20

**Authors:** Lihui Liu, Chao Wang, Sini Li, Yan Qu, Pei Xue, Zixiao Ma, Xue Zhang, Hua Bai, Jie Wang

**Affiliations:** ^1^ National Cancer Center/National Clinical Research Center for Cancer/Cancer Hospital, Chinese Academy of Medical Sciences and Peking Union Medical College, Beijing, China; ^2^ Sleep Medicine Center, West China Hospital, Sichuan University, Chengdu, China; ^3^ State Key Laboratory of Molecular Oncology, Department of Medical Oncology, National Cancer Center/National Clinical Research Center for Cancer/Cancer Hospital, Chinese Academy of Medical Sciences and Peking Union Medical College, Beijing, China

**Keywords:** *ERO1L*, tumor microenvironment, biomarker, immunotherapy, lung adenocarcinoma

## Abstract

**Background:**

The endoplasmic reticulum oxidoreductin-1-like (*ERO1L*) gene encodes an endoplasmic reticulum luminal localized glycoprotein known to associated with hypoxia, however, the role of *ERO1L* in shaping the tumor immune microenvironment (TIME) is yet to be elucidated in lung adenocarcinoma (LUAD).

**Methods:**

In this study, raw datasets (including RNA-seq, methylation, sgRNA-seq, phenotype, and survival data) were obtained from public databases. This data was analyzed and used to explore the biological landscape of *ERO1L* in immune infiltration. Expression data was used to characterize samples. Using gene signatures and cell quantification, stromal and immune infiltration was determined. These findings were used to predict sensitivity to immunotherapy.

**Results:**

This study found that *ERO1L* was significantly overexpressed in LUAD in comparison to normal tissue. This overexpression was found to be a result of hypomethylation of the *ERO1L* promoter. Overexpression of *ERO1L* resulted in an immune-suppressive TIME *via* the recruitment of immune-suppressive cells including regulatory T cells (T_regs_), cancer associated fibroblasts, M2-type macrophages, and myeloid-derived suppressor cells. Using the Tumor Immune Dysfunction and Exclusion (TIDE) framework, it was identified that patients in the *ERO1L* ^high^ group possessed a significantly lower response rate to immunotherapy in comparison to the *ERO1L* ^low^ group. Mechanistic analysis revealed that overexpression of *ERO1L* was associated with the upregulation of JAK-STAT and NF-κB signaling pathways, thus affecting chemokine and cytokine patterns in the TIME.

**Conclusions:**

This study found that overexpression of *ERO1L* was associated with poor prognoses in patients with LUAD. Overexpression of *ERO1L* was indicative of a hypoxia-induced immune-suppressive TIME, which was shown to confer resistance to immunotherapy in patients with LUAD. Further studies are required to assess the potential role of *ERO1L* as a biomarker for immunotherapy efficacy in LUAD.

## Introduction

Lung cancer is a leading cause of cancer-related mortality and lung adenocarcinoma (LUAD) accounts for approximately 50% of all reported cases (1). In recent years, as precision medicine is becoming a reality, LUAD treatments have gradually evolved from empirical chemotherapy to personalized therapies. Immunotherapies, which have the advantage of high efficiency, long duration, and low toxicity, have led to a paradigm shift in cancer treatment. Immunotherapy has become the standard of care for advanced LUAD. However, widespread usage of immunotherapy is limited and drug resistance is increasingly reported (2, 3). As a result, the identification of biomarkers to enable patient selection is urgently required.

There is a growing body of evidence supporting the theory that the tumor immune microenvironment (TIME) plays a crucial role in the response to immunotherapy. The TIME comprises a series of infiltrating cells, such as neoplastic cells, immune cells, endothelial cells, fibroblasts. Different infiltration components are associated with different clinical outcomes. Based on immune score, recent research has classified TIME into three subtypes: immune-inflamed (I-I TIME), immune-desert (I-D TIME), and immune-excluded (I-E TIME) (4). Patients with I-I TIME are frequently reported to be infiltrated with an abundance of inflammatory cells. This indicates that they will have a significant clinical response to immune checkpoint inhibitor (ICI) therapy. Contrastingly, I-D TIME and I-E TIME are both be considered noninflamed TIME. As such, these patients are rarely responsive to ICI therapy (5). The differential responses of these subtypes present the need to develop individualized treatment strategies. However, two key challenges remain. Firstly, determination of the threshold for an inflamed or noninflamed TIME. And secondly, the lack of appropriate biomarkers that are able to distinguish TIME subtypes (6).

The endoplasmic reticulum oxidoreductin-1-like (*ERO1L*) gene, which is located on chromosome 14 in humans, is considered to be the primary source of the endoplasmic reticulum. ERO1L is an endoplasmic reticulum luminal localized glycoprotein which favors disulfide bond formation *via* the selective oxidization from protein disulfide isomerases (7). Hypoxia is a hallmark of the tumor microenvironment and is reported in the majority of tumors overexpressing *ERO1L (* 8). Hypoxic stress has been described to cause immunosuppression by controlling angiogenesis. This is predicted to result in resistance to ICI therapy (9). What’s more, *ERO1L* is known to promote programmed death-ligand 1 (PD-L1) expression by increasing the expression of hypoxia-inducible factor1α (HIF-1α) and subsequently facilitating oxidative protein folding within PD-L1. Ultimately, this results in immune escape (10). The role of *ERO1L* in the crafting of the tumor immunological microenvironment is yet to be elucidated.

In this study, the association between *ERO1L* expression and TIME was investigated in LUAD. Bioinformatics techniques including cell quantification algorithms and gene expression profiling were used. This study identified that the overexpression of *ERO1L* is a feature in an immune-suppressive TIME. This provided insight into the potential association between *ERO1L* and tumor-immune interactions.

## Materials and Methods

### mRNA and Protein Expression Analyses of *ERO1L* Using Public Databases

The *ERO1L* mRNA expression in pan-cancer was analyzed in the Oncomine database (www.oncomine.org) with the following thresholds: *p*-value of 0.001, a fold change of 1.5, and a top 10% gene ranking (11). The expression data belonging to four datasets (GSE7670, GSE31210, GSE32863, and GSE19188) were downloaded from the GEO database (https://www.ncbi.nlm.nih.gov/geo/). Expression profiles were normalized by z-scores of log_2_(count+1). Using the *limma* package in R-4.0.3, batch normalization was also completed. Protein expression levels were analyzed using the HPA database (http://www.proteinatlas.org). Antibodies used in the HPA database included HPA026653 (Sigma-Aldrich), HPA030053 (Sigma-Aldrich) and CAB034294 (Santa Cruz Biotechnology). These three antibodies were all validated by orthogonal method (antibody staining mainly consistent with RNA expression data across 41 tissues) and by independent antibodies (protein distribution across 45 tissues similar between the independent antibodies HPA026653 and HPA030053).

### TCGA Database Analysis

TCGA LUAD data was downloaded from the UCSC Xena database (http://xena.ucsc.edu/). This data included information on: RNA-seq (HTSeq-FPKM), DNA methylation (Illumina Human Methylation 450), and clinical profiles (including both phenotype and survival data). Expression levels were normalized using the z-score of log_2_(FPKM+1) to exclude potential bias. Patients were assigned into *ERO1L*-high and *ERO1L*-low groups based on the median expression value according to the RNA sequencing data. In terms of methylation analysis, we included 18 methylation sites (**Table S4**). These sites were mapped to the *ERO1L* gene using the UCSC Genome Browser HG19 RefSeq database (http://genome.ucsc.edu). The methylation level of each CpG site was recorded as a β value. This value indicated the ratio of the methylated signal intensity over the sum of the methylated and unmethylated intensities at each locus. Each locus with an average β-value of less than 0.20 was considered a hypomethylation site. In addition, Kaplan-Meier survival curves were plotted to show differences in survival time. For this data, log-rank *p*-values reported in the *survival* package in R-4.0.3 were used to determine statistical significance. Path analysis was performed in the *ggalluvial* in R-4.0.3. Visualization was performed using the OriginPro 2019b software (version 9.6.5.169).

### Organoid Culture

Mouse lung tissue was carefully dissected avoiding other tissue contamination, then minced with surgical scalpels and incubated in Trypsin (Gibco) at 37°C for 40-50 min. The digestion was terminated with 10% serum (Gibco), and digested tissues were filtered with a 70 μm cell strainer in order to filter out debris that had not been fully digested. Cells were resuspended with Matrigel (Corning) and plated in 48 well plates. Then the Matrigel was solidified in an incubator at 37°C for 15-20 min and overlaid with 150-200 μL medium. Organoid medium contains advanced DMEM/F12, FGF 100 ng/mL, EGF 10 ng/mL, B27 supplement (2 X final), N2 supplement (1 X final), Noggin 100 ng/mL, RSPO-1 (10% final), Wnt-3a (10% final), Y27632 10 μM,A83-01 10 μM, Glutamax (1 X final) and Penstrep (1 X final). Cultures were kept at 37°C, 5% CO2 in an incubator and the medium was exchanged every 4-8 days according to the number of spheres. For passaging, the Matrigel containing organoids was dissolved in 3-5 mL TrypLE at 37°C for 10 min, and pipetted vigorously (80-100 times) to dissociate organoids into single cells. Cells were filtered with 70 μm cell strainer, centrifuged at 1,500 rpm for 10 min and resuspended with Matrigel.

### Organoid Infection

Organoids were dissociated into single cells as described above, resuspended with 200 μL medium ae well as 2-3 mL virus particles, and added polybrene to 1 X final. After being centrifuged at 2,000 rpm, 37°C for 1 hour, the cells were then incubated at 4°C, 5% CO_2_ for 2-3 hours. Finally, an organoid culture was performed as described above.

### Co-Expression Module Identification and Pathway Analysis

Firstly, genes which were co-expressed were identified in the Oncomine and TCGA databases respectively. By overlapping the results from these two databases, we identified a co-expression module consisting of 17-genes. STRING (version 10.5) was used to construct a protein-protein interaction (PPI) network. The 17 genes within the module were subjected to pathway enrichment analysis using DAVID (https://david-d.ncifcrf.gov/) (12). Results were visualized using the *Hmisc and ggplot2* packages in R-4.0.3. Gene set enrichment analysis (GSEA) was performed using the GSEA software (version 4.1.0) and Broad’s GSEA algorithm (13).

### Immune Infiltration Analysis

The relationship between *ERO1L* expression levels and immune infiltration was initially determined using the TIMER2.0 database (http://timer.comp-genomics.org). The TIMER2.0 database utilizes *immunedeconv*, which is an R package integrating six state-of-the-art algorithms. These algorithms include: TIMER, xCell, MCP-counter, CIBERSORT, EPIC, and quanTIseq (14). These algorithms were systematically benchmarked, and each was found to have unique properties and strengths. *ERO1L* expression was analyzed in the presence of seven types of immune infiltrating cells, including B cells, CD4^+^ T cells, CD8^+^ T cells, NK cells, macrophages, CAFs, and MDSCs. During immune infiltration analysis, adjustments were also made for tumor purity. The online tool CIBERSORTx (https://cibersortx.stanford.edu) was used to estimate different immune cell proportions (15).

Correlation analysis between the expression of *ERO1L*, immune cell markers and cytokines as well as chemokines was also performed using the TIMER2.0 database, specifically using the Gene_Corr module. The functionality of this module allows users to uncover the co-expression pattern of genes across TCGA cancer types. When provided with one initial gene of interest and up to 20 other genes, the TIMER2.0 database generates a heatmap table of Spearman’s correlation of gene expression between the gene of interest and the other input genes. After adjustment for tumor purity, a Spearman’s ρ >0 with *p*-value <0.05 was considered as a positive correlation and a Spearman’s ρ <0 with *p*-value <0.05 was considered as a positive correlation. Secondary confirmation of correlation analysis was performed using GEPIA (http://gepia.cancer-pku.cn) (16). Tertiary confirmation of correlation analysis was performed using TCGA expression data from the GEPIA database (http://gepia.cancer-pku.cn/index.html).

### Single-Cell Sequencing Analysis

Processed gene expression data was download from the GEO database (GSE99254). This project consists of deep single-cell transcriptome data with complete T cell receptor information, which identified multi-dimensional characteristics of infiltrating lymphocytes (17). Single-cell transcriptome data was analyzed based on t-SNE dimension reduction using the R package *Rtsne*. Additionally, the Tumor Immune Single-cell Hub (TISCH) database was used to analyze the correlations between *ERO1L* expression and infiltrating immune cells (18). TISCH is a scRNA-seq database focusing on the tumor microenvironment. This database includes 79 datasets and 2,045,746 cells. TISCH provides detailed cell-type annotation at the single-cell level, enabling detailed exploration of the tumor microenvironment across various different cancer types.

### Immunotherapy Response Prediction

In the first instance, in order to estimate the presence of the various immune cell populations in the LUAD tissues, the R package *ESTIMATE* was used. Estimation of STromal and Immune cells in MAlignant Tumor tissues using Expression data (ESTIMATE) is a tool that predicts tumor purity *via* the use of gene signatures. The tool calculates three scores, including stromal score, which predicts the presence of stromal cells in tumor bulk; immune score, which infers the levels of immune cells infiltration in tumor tissue; and estimate score, which estimates tumor purity. Subsequently, the R package *MCPcounter* was used to develop a more detailed idea of the level of immune cell infiltration. Microenvironment Cell Populations-counter (MCPcounter) is a quantification method that determines the relative abundance of an immune cell in heterogeneous tissues. This method uses marker genes optimized for interrogating microarray data (19). In order to predict the response to immune checkpoint blockade, Tumor Immune Dysfunction and Exclusion (TIDE) score was employed. TIDE is a computational method used to model two primary mechanisms of tumor immune evasion. These mechanisms are: the induction of T cell dysfunction in tumors with high infiltration of cytotoxic T lymphocytes (CTL) and the prevention of T cell infiltration in tumors with low CTL level (20, 21). Using this framework and RNA-Seq tumor expression profiles, TIDE can predict the outcomes of non-small cell lung cancer (NSCLC) patients treated with first-line anti-PD1 or anti-CTLA4 more accurately than other biomarkers such as PD-L1 levels and mutational load.

### Statistical Analysis

Chi-squared and Fisher’s exact tests were used to investigate the significance of the correlation of *ERO1L* expression with clinicopathological features in LUAD patients. Analysis was performed using SPSS (version 23.0). ANOVA was used to identify the *ERO1L* expression levels in different datasets (22). The correlation of gene expression was evaluated using Spearman’s correlation coefficient (23). A *p* value <0.05 was considered statistically significant.

## Results

### Quantification of *ERO1L* mRNA Expression in Pan-Cancer

**Figure 1:**
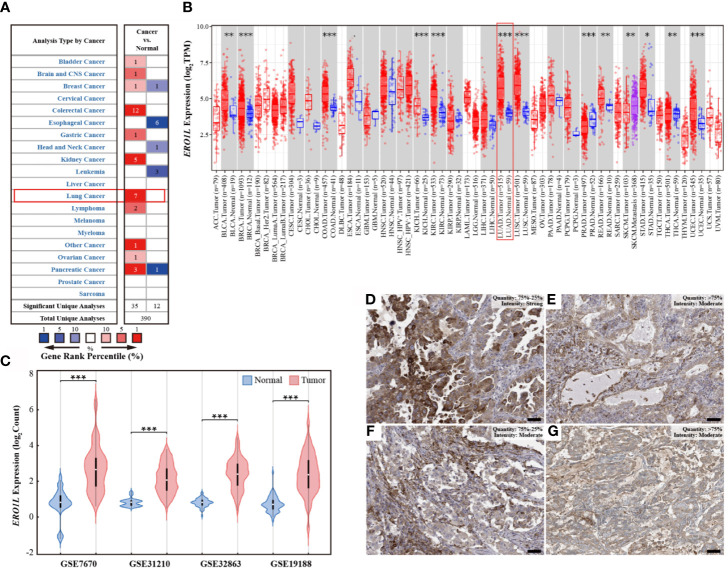
*ERO1L* expression levels in pan-cancer and LUAD. **(A)** Expression levels of *ERO1L* mRNA in pan-cancer, compared with normal tissues in the Oncomine database. The number in each cell denotes the number of datasets. **(B)** Expression levels of *ERO1L* mRNA in pan-cancer, compared with normal tissue from the TCGA database. **(C)** Expression levels of *ERO1L* mRNA in LUAD across four GEO datasets. **(D-G)** Expression levels of *ERO1L* protein in four patients with LUAD from the HPA database. Scale bar: 50 µm. *p < 0.05; **p < 0.01; ***p < 0.001.

In order to determine the mRNA expression profile of *ERO1L* in pan-cancer, expression levels of *ERO1L* in the Oncomine database were analyzed. Comparisons of mRNA expression levels of *ERO1L* in pan-cancers versus normal tissue identified nine types of cancer in which *ERO1L* mRNA expression levels were elevated. These types of cancer included bladder, brain, central nervous system, colorectal, gastric, kidney, lung, lymphoma, ovarian, and pancreatic cancer. In addition, three types of cancer in which *ERO1L* mRNA expression levels were diminished were identified. These types of cancer included esophageal cancer, head and neck cancer, and leukemia (**Figure 1A**). What’s more, lung cancer was found to be associated with a significantly higher expression level of *ERO1L* in comparison to normal tissue. The expression levels of *ERO1L* were increased in seven datasets while no dataset possessed decreased levels of *ERO1L*.

To further investigate the mRNA expression levels of *ERO1L* in pan-cancers, RNA-sequencing data from The Cancer Genome Atlas (TCGA) program was analyzed. Interestingly, it was discovered that *ERO1L* mRNA expression levels were somewhat increased in pan-cancers in comparison to normal tissues (**Figure 1B**). This finding is consistent with our analysis of data from the Oncomine database, which revealed significantly elevated expression levels of *ERO1L* in lung adenocarcinoma (LUAD; *p <*0.001) and in lung squamous cell carcinoma (LUSC; *p <*0.001).

### Expression Profiles of *ERO1L* mRNA and Protein in LUAD

In order to study the mRNA expression levels of *ERO1L* in LUAD, further analysis was performed on datasets from the Gene Expression Omnibus (GEO) database. In this analysis, four datasets (GSE7670, GSE31210, GSE32863, and GSE19188) comprising a total of 526 samples were included. This included 356 tumoral and 170 paired normal biopsies (**Table S1**). After normalizing the expression profile, it was identified that *ERO1L* mRNA expression levels in LUAD was significantly elevated in comparison to normal tissues. This was observed in all of the datasets analyzed. Interestingly, expression fold changes ranged from 2.8 to 4.1 times (**Figures 1C** and **S1A**). In addition, the mRNA expression of *ERO1L* in LUAD was investigated using the TCGA program. Similarly, significantly elevated expression levels of *ERO1L* mRNA were observed in LUAD in comparison to normal tissues. This was observed when the analysis was performed using both TCGA program and the Genotype-Tissue Expression (GTEx) program (**Figure S1B**).

These elevated *ERO1L* mRNA expression levels were confirmed in LUAD. As a logical next step, the protein expressions of *ERO1L* in LUAD were then investigated. Analysis using The Human Protein Atlas (HPA) program revealed that ERO1L was positively detected *via* immunohistochemistry (IHC) staining in patients with LUAD. Eleven patients with LUAD were identified in the HPA database, all of these patients possessed positive ERO1L protein expression (**Figures 1D–G**). Out of these patients, the intensity of IHC staining is as follows: three patients were associated with strong intensity, six with moderate, and two with weak intensities of IHC staining (**Table S2**).

### Overexpression of *ERO1L* Is Associated With a Poorer Prognosis in LUAD

**Figure 2:**
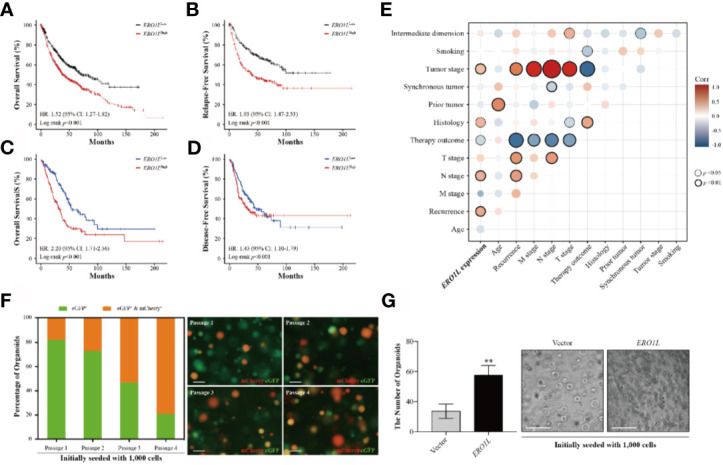
Overexpression of *ERO1L* predicts a poorer prognosis in LUAD. **(A, B)** Survival analysis comparing low and high expression levels of *ERO1L* in the Kaplan-Meier plotter database. Overexpression of *ERO1L* was correlated with significantly poorer overall survival **(A)** and relapse-free survival **(B)**. Overexpression of *ERO1L* was correlated with significantly poorer overall survival **(C)** and disease-free survival **(D)**. **(E)** Association between the expression of *ERO1L* and clinicopathological characteristics in patients with LUAD. **(F)** Fluorescence images of *ERO1L*-overexpressed organoids show an increase in the number of chimeric organoids over passaging. **(G)** Quantification of *ERO1L* upregulated organoids relative to the average number of organoid spheres in four random fields. **p < 0.01.

In order to study the correlation between *ERO1L* expression and prognosis in LUAD patients, six cohorts of patients were obtained from the PrognoScan database (**Table S3**). Via the analysis of hazard ratios (HR) and 95% confidence intervals (CI), four cohorts of LUAD patients (HLM, Nagoya, UM, and NCCRI) with high expression of *ERO1L* were identified. This high expression of *ERO1L* was associated with worsened prognoses in these patients as measured by overall survival (OS) and recurrent-free survival (RFS). Similarly, analysis was performed on survival data from the Kaplan-Meier plotter database. This is based on the Affymetrix microarrays with probe ID 218498_s_at for the *ERO1L* gene. These results showed consistently that overexpression of *ERO1L* was associated with worse prognoses in patients with LUAD in terms of OS (HR: 1.52, 95% CI: 1.27-1.82; **Figure 2A**) and RFS (HR: 1.93, 95% CI: 1.47-2.53; **Figure 2B**).

The following hypothesis was proposed: *ERO1L* is a potential biomarker in patients with LUAD. To investigate this hypothesis, survival analysis was applied to the RNA-sequencing data obtained from the TCGA program. This analysis revealed that there was a significant correlation between *ERO1L* overexpression, shorter overall survival (HR: 2.20, 95% CI: 1.71-2.56; **Figure 2C**) and disease-free survival (HR: 1.43, 95% CI: 1.10-1.79; **Figure 2D**). Interestingly, *via* correlation and multiple linear regression analysis, it was found that the expression level of *ERO1L* correlated with specific clinicopathological characteristics in LUAD patients (**Figure 2E**). As shown in **Table 1**, *ERO1L* overexpression was significantly correlated with tumor recurrence, pathologic N stage, primary treatment outcome, tumor histology, and tumor stage.

**Table 1:** Correlation between ERO1L and clinicopathological characteristics in patients with lung adenocarcinoma.

Clinicopathological Characteristics	*ERO1L* Expression		*p*-value
	Low	High	
**Age (year)**	65.13 ± 10.10	63.86 ± 10.74	0.262
**Smoking status**			
Years smoked	31.65 ± 13.23	28.31 ± 12.71	0.168
Cigarettes/day	2.23 ± 1.51	2.19 ± 1.34	0.855
**Tumor dimension**			
Intermediate dimension	0.79 ± 0.33	0.80 ± 0.33	0.832
Longest dimension	1.24 ± 0.54	1.22 ± 0.61	0.791
Shortest dimension	0.39 ± 0.18	0.38 ± 0.15	0.593
**Tumor recurrence**	91 (28.4%)	40 (43.0%)	0.008
**Tumor stage**			0.026
Stage I	211 (59.2%)	49 (45.8%)	
Stage II	79 (22.1%)	32 (29.9%)	
Stage III	51 (14.3%)	22 (20.6%)	
Stage IV	16 (4.4%)	4 (3.7%)	
**T stage**			0.574
T1	131 (36.7%)	32 (29.9%)	
T2	183 (51.3%)	61 (57.0%)	
T3	31 (8.7%)	9 (8.4%)	
T4	12 (3.4%)	5 (4.7%)	
**N stage**			0.020
N0	248 (69.3%)	60 (55.6%)	
N1	66 (18.4%)	27 (25.0%)	
N2	44 (12.3%)	20 (18.5%)	
N3	0 (0.0%)	1 (0.9%)	
**M stage**			0.746
M0	227 (93.8%)	73 (94.8%)	
M1	15 (6.2%)	4 (5.2%)	
**Primary treatment outcome**			0.040
Progressive disease	44 (15.7%)	22 (28.6%)	
Stable disease	27 (9.6%)	4 (5.2%)	
Partial remission	4 (1.4%)	0 (0.0%)	
Complete remission	205 (73.2%)	51 (66.2%)	
**Tumor histology**			0.028
Adenocarcinoma	203 (56.5%)	76 (70.4%)	
Adenocarcinoma with mixed subtypes	80 (22.3%)	15 (13.9%)	
Acinar cell carcinoma	17 (4.7%)	3 (2.8%)	
Bronchiolo-alveolar carcinoma, non-mucinous	17 (4.7%)	1 (0.9%)	
Papillary adenocarcinoma	16 (4.5%)	4 (3.7%)	
Mucinous adenocarcinoma	15 (4.2%)	2 (1.9%)	
Bronchiolo-alveolar carcinoma, mucinous	4 (1.1%)	1 (0.9%)	
Micropapillary carcinoma	2 (0.6%)	0 (0.0%)	
Bronchiolo-alveolar adenocarcinoma	2 (0.6%)	1 (0.9%)	
Solid carcinoma	2 (0.6%)	4 (3.7%)	
Signet ring cell carcinoma	1 (0.3%)	0 (0.0%)	
Clear cell adenocarcinoma	0 (0.0%)	1 (0.9%)	
**Prior malignancy**	61 (17.0%)	15 (13.9%)	0.444
**Synchronous malignancy**	7 (2.2%)	2 (2.0%)	0.943

Furthermore, we applied an organoid model to study the biological function of *ERO1L*. We designed a protocol for organoid infection through dissociating organoid spheres into single cells and then co-culturing with virus particles. By introducing cDNA encoding *ERO1L* (labeled with mCherry) into organoids (labeled with eGFP), we obtained *ERO1L*-overexpressed organoids labeled in different colors. Organoids would turn red when transduced with cDNA, indicating the overexpression of *ERO1L* (**Figure 2F**). Using this, we detected infected organoids and calculated the ratios of chimeric organoids from passage 1 to 4. The initial percentage of chimeric organoids was about 16%, and it gradually increased to 75% after passaging three times (**Figure 2F**). Besides, the doubling time of organoid cells decreased from five to six days at passage 1 to two to two and a half days at passage 4. Transduction of cDNA of *ERO1L* in organoids was also performed. One thousand cells in each group were seeded and cultured for fourteen days (passage 2). Sphere formation was enhanced from cells overexpressing *ERO1L* compared with the control (**Figure 2G**). Hence, we concluded that organoids with *ERO1L* overexpression gradually gained an advantage in development, which could be extended over time.

### Regulation of *ERO1L* mRNA Level *via* Promoter Methylation

**Figure 3:**
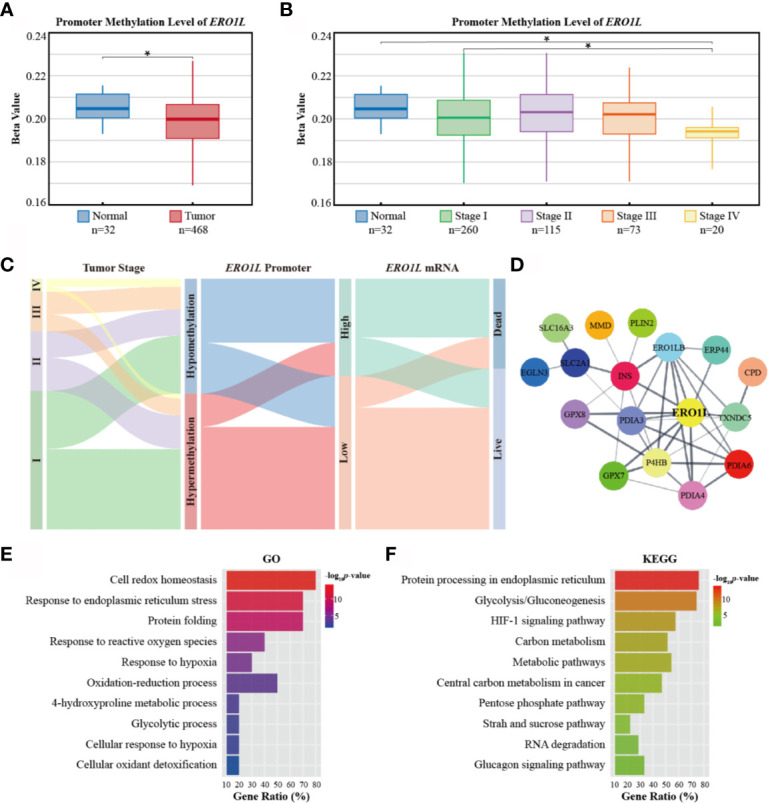
Promoter methylation of *ERO1L* and functional annotation of the *ERO1L* co-expression module. **(A, B)** Analysis of *ERO1L* methylation in the TCGA database. Promoter methylation levels of *ERO1L* in tumor and normal tissues **(A)** according to tumor stage **(B)**. **(C)** Path analysis in patients with LUAD across tumor stage, promoter methylation, mRNA expression, and survival status. The line represents the group; the width of the lines represents the number of patients transferred from one state to another (n=468). **(D)** PPI networks of the *ERO1L* co-expression module. **(E, F)** Functional annotation and pathway enrichment of the *ERO1L* co-expression module. Top 10 terms of GO annotation **(E)** and KEGG pathway **(F)**. *p < 0.05.

In order to elucidate the mechanism underlying *ERO1L* expression, promoter methylation levels of *ERO1L* were investigated in 503 samples. This was performed *via* analysis of methylation profiles (Illumina Human Methylation 450) from the TCGA program (**Table S4**). A significant decrease in the methylation level of the *ERO1L* was identified in the promoter region in LUAD tissues in comparison to normal tissues (**Figure 3A**). Tumor stage subgroup analysis revealed that levels of *ERO1L* promoter methylation were most significantly decreased in stage IV patients (**Figure 3B**). In addition, correlation analysis revealed a significant negative correlation between mRNA levels and methylation levels of *ERO1L* (Spearman’s ρ: -0.25, p <0.001). This confirmed that *ERO1L* mRNA expression was regulated by promoter methylation in LUAD patients (**Figure S1C**).

In order to analyze the clinical outcomes associated with methylation and expression of *ERO1L*, datasets of 468 patients in the TCGA database were analyzed. These datasets all contained data corresponding to methylation, expression, and survival profiles. Path analysis *via* a Sankey diagram was performed. This quantified and visualized the transitions with various lines and widths, and described paths and patterns across tumor stages, promoter methylation levels, *ERO1L* mRNA expression, and survival status (**Figure 3C**). As a result, it was determined that hypomethylation of the *ERO1L* promoter potentially induced overexpression of *ERO1L* mRNA and finally led to poor prognoses in individuals with LUAD. This pattern was observed more significantly in patients with advanced stages of cancer (**Figure S2A**). This data is consistent with the survival analysis based on *ERO1L* expression. This provides compelling evidence that *ERO1L* is associated with poor prognoses in patients with LUAD.

### Co-Expression Module of *ERO1L* in LUAD

In order to investigate proteins that were in close relationship to *ERO1L*, a co-expression analysis of expression data from the TCGA program and Oncomine database was conducted. Via overlapping co-expression results and module mining, 29 proteins were found to be closely related to *ERO1L* (**Figure S2B**). This included ERO1LB, GPX7, GPX8, P4HB, INS, PDIA3, PDIA4, PDIA6, TXNDC5, and ERP44 among others. (**Table S5**). Based on these results, the protein-protein interaction (PPI) network of the *ERO1L* co-expression module was created (**Figure 3D**, **Table S6**). Furthermore, the biological functions of the module were investigated by Gene Otology (GO) analysis (**Figure 3E**) and Kyoto Encyclopedia of Genes and Genomes (KEGG) analysis (**Figure 3F**). Interestingly, it was found that these genes showed strong associations with significant processes such as response to endoplasmic reticulum stress, response to reactive oxygen species, oxidation-reduction process, and glycolytic process. Integrally, this module was shown to be closely related to hypoxia responses as well as the HIF-1 signaling pathway. These might also play a role in shaping the TIME.

### Correlations Between *ERO1L* Expression and Immune Cell Markers

**Figure 4:**
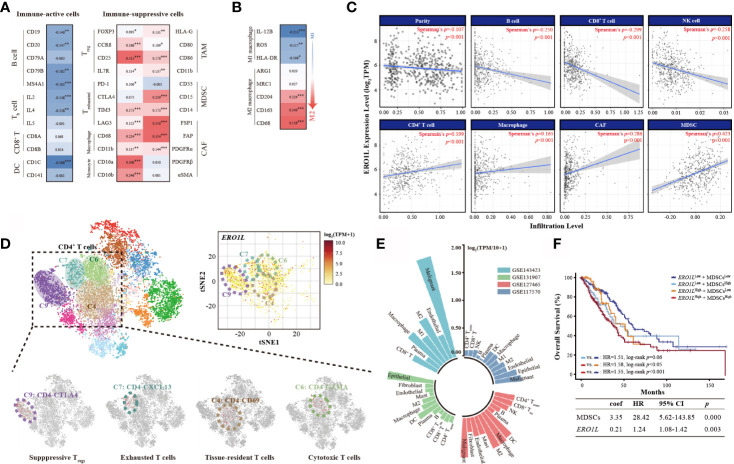
Overexpression of *ERO1L* shapes an immune-suppressive tumor microenvironment. **(A)** Correlations between *ERO1L* and the immune cell markers. **(B)** *ERO1L* expression correlates with macrophages polarization. **(C)** *ERO1L* expression was significantly negatively correlated with infiltrating levels of B cells, CD8+ T cells, and NK cells and significantly positively correlated with infiltrating levels of CD4+ T cells, macrophages, CAFs, and MDSCs. **(D)** The intrinsic heterogeneity of CD4^+^ T cells according to *ERO1L* expression as determined *via* single-cell sequencing data. Each dot corresponds to a single cell and is colored according to the cell cluster. The color density indicates the expression of *ERO1L*. **(E)** Summary of four single-cell sequencing datasets according to correlations with *ERO1L*. Different datasets are labeled in different colors. **(F)** The Cox proportional hazards model is constructed according to expressions of *ERO1L* and MDSC. *p < 0.05; **p < 0.01; ***p < 0.001.

To first understand the relationship between *ERO1L* and infiltrating immune cells, a correlation analysis across *ERO1L* and markers for immune cells was performed. These biomarkers are widely used for the purpose of immune cell characterization (**Table 2**). *ERO1L* expression showed strong correlations with markers for infiltrating lymphocytes including regulatory T cells (T_regs_), exhausted T cells, macrophages, tumor-associated macrophages (TAMs), myeloid-derived suppressor cells (MDSCs), and cancer-associated fibroblasts (CAFs), thus indicating infiltrations of immune-suppressive cells are mediated by *ERO1L* signaling (**Figure 4A**). Interestingly, *ERO1L* expression was shown to be positively correlated with the phenotype of M2-type macrophages while negatively correlated with the phenotype of M1-type macrophages. This implies that overexpression of *ERO1L* could indicate the polarization of M1-type to M2-type macrophage (**Figure 4B**).

**Table 2:** Correlations between *ERO1L* and gene markers of infiltrating immune cells.

Cell Type	Gene Marker	Without Adjusted		Purity Adjusted	
		Correlation	*p*-value	Correlation	*p*-value
**B cell**	CD19	-0.065	0.140	-0.140	**
	CD20	-0.119	**	-0.147	**
	CD79A	-0.023	0.606	-0.083	0.067
	CD79B	-0.103	*	-0.182	***
	MS4A1	-0.108	*	-0.183	***
**CD8^+^ T cell**	CD8A	0.098	0.262	0.063	0.163
	CD8B	0.046	0.296	0.014	0.761
**Th1**	IL-2	-0.016	**	-0.148	***
**Th2**	IL-4	-0.136	**	-0.138	**
	IL-5	-0.012	0.788	-0.001	0.981
**T_reg_**	FOXP3	0.123	**	0.093	*
	CCR8	0.175	***	0.168	***
	CD25	0.312	***	0.313	***
	IL7R	0.140	**	0.114	*
**T cell exhausted**	PD-1	0.133	**	0.100	*
	CTL4	0.107	*	0.075	0.097
	TIM3	0.194	***	0.173	***
	LAG3	0.145	***	0.123	**
**DC**	CD1C	-0.235	***	-0.269	***
	CD141	-0.065	0.143	-0.081	0.073
**Macrophage**	CD68	0.241	***	0.224	***
	CD11b	0.152	***	0.137	**
**M1**	NOS2	0.070	0.112	0.043	0.345
	ROS	-0.096	*	-0.117	**
	IL-12B	-0.179	***	-0.213	***
	HLA-DR	-0.069	0.117	-0.108	*
**M2**	ARG1	0.018	0.676	0.020	0.664
	MRC1	0.046	0.295	0.027	0.552
	CD68	0.241	***	0.224	***
	CD163	0.256	***	0.248	***
	CD204	0.181	***	0.156	***
**TAM**	HLA-G	0.154	***	0.131	**
	CD80	0.130	**	0.109	*
	CD86	0.193	***	0.178	***
	CD11b	0.152	***	0.137	**
**Monocyte**	CD14	0.183	***	0.173	***
	CD16a	0.315	***	0.308	***
	CD16b	0.245	***	0.246	***
**MDSC**	CD11b	0.152	***	0.137	**
	CD33	0.021	0.639	-0.005	0.905
**PMN-MDSC**	CD15	0.267	***	0.259	***
**M-MDSC**	CD14	0.183	***	0.173	***
**CAF**	FSP1	0.308	***	0.318	***
	FAP	0.314	***	0.314	***
	PDGFRα	0.158	***	0.144	**
	PDGFRβ	0.086	0.052	0.058	0.200
	αSMA	0.105	*	0.081	0.072

TAM, tumor associated macrophage; MDSC, myeloid-derived suppressor cell; PMN-MDSC, polymorphonuclear myeloid-derived suppressor cell; M-MDSC, monocytic myeloid-derived suppressor cell; CAF, cancer-associated fibroblast; Cor., R value of Spearman’s correlation. *p < 0.05; **p < 0.01; ***p < 0.001.

### *ERO1L* Mediated Immune-Suppressive Tumor Microenvironment Shaping

To confirm whether *ERO1L* expression impacts the TIME, the coefficients of *ERO1L* expression and TIME infiltrations were calculated in the Tumor IMune Estimation Resource 2.0 (TIMER 2.0) database. In relation to tumor-infiltrating lymphocytes, it was found that immune-active cells including B cells (Spearman’s ρ=-0.250, *p <*0.001), CD8^+^ T cells (Spearman’s ρ=-0.299, *p <*0.001), and NK cells (Spearman’s ρ=-0.258, *p <*0.001) correlated negatively with *ERO1L* expression. After adjustments to account for tumor purity, immune-suppressive cells CAFs (Spearman’s ρ=0.286, *p <*0.001) and MDSCs (Spearman’s ρ=0.423, *p <*0.001) were shown to be positively correlated with *ERO1L* expression (**Figure 4C**).

As a positive correlation was observed between *ERO1L* and CD4^+^ T cells (Spearman’s ρ=0.199, *p <*0.001), intrinsic CD4^+^ T cell heterogeneity was further investigated *via* analysis of single-cell sequencing data from dataset GSE99254. Dimensional reduction analysis (t-SNE) applied to the expression data showed that *ERO1L* was highly expressed in most CD4^+^ T cell clusters. This was consistent with our previous findings (**Figure 4D**). The clusters of CD4-CTLA4 (cluster C9) and CD4-CXCL13 (cluster C7), representing suppressive T_regs_ and exhausted T cells respectively, showed the highest *ERO1L* expression levels. To further confirm *ERO1L* expression across infiltrating cells in TIME, single-cell sequencing data from four projects (GSE7670, GSE31210, GSE32863, and GSE19188) was analyzed. Results showed that *ERO1L* expression closely correlated with infiltrating cell levels including B cells, T cells, NK cells, endothelial cells, macrophages, monocytes, MDSCs, and CAFs (**Figure 4E**, **Figure S2C**). *ERO1L* was more closely associated with the phenotype of a M2 macrophage than a M1 macrophage, which was consistent with our previous findings.

Of note, MDSCs are known to play a key role in immunosuppression in various cancer types. In recent years, increasing evidence has highlighted MDSCs as a major driver behind the immunosuppressive tumor microenvironment. As C/EBPβ and c-Rel have been implicated in MDSC expansion, C/EBPβ and c-Rel expressions were examined. Consistent with previous findings, it was identified that both C/EBPβ and c-Rel were significantly positively correlated with *ERO1L* expression (C/EBPβ: Spearman’s ρ=0.144, *p <*0.001; c-Rel: Spearman’s ρ=0.201, *p <*0.001). This supports the notion that *ERO1L* signaling potentially results in the accumulation of functional MDSCs (**Figure S2D**). Based on the strong correlation observed between *ERO1L* and MDSCs, a survival analysis was performed by constructing a Cox proportional hazards model according to expression profiles of *ERO1L* and MDSC (**Figure 4F**). Results revealed that patients exhibiting low levels of both *ERO1L* and MDSCs experienced a significantly better OS in comparison to those with simultaneously high levels of *ERO1L* and MDSCs (HR:1.55, 95% CI: 1.12-1.84, log-rank *p <*0.001). This indicates that the combination of high levels of *ERO1L* and MDSC expression can predict poor prognoses in patients with LUAD. In tumors where the high expression level of *ERO1L* was a result of copy number variations including gain and amplification compared with deletion or normal diploid, significant differences were also noted. In specific cases there a decrease in CD8^+^ T cells and an increase of CAFs and macrophages (**Figures S2E, F**). Taken together, these results indicate that *ERO1L* overexpression is closely related to infiltration of immune-suppressive cells and the deficiency of immune-active cells, thus shaping an immunosuppressive TIME.

### *ERO1L* Overexpression Can Potentially Predict Immunotherapy Resistance

Based on the notion that *ERO1L* overexpression shaped an immune-suppressive TIME, it was hypothesized that high levels of *ERO1L* might also lead to immunotherapy resistance. Given that the MC-38 cell line was sensitive to ICI treatment while the LLC and A549 cell lines were relatively insensitive to ICI treatment, we first performed Western blotting to examine the expression levels of ERO1L protein across these cell lines. Results showed that ERO1L protein was overexpressed in the LLC and A549 cell lines while downregulated in the MC-38 cell line (**Figure S3A**), which was consistent with our hypothesis that overexpression of *ERO1L* might be associated with resistance to ICI treatment.

**Figure 5:**
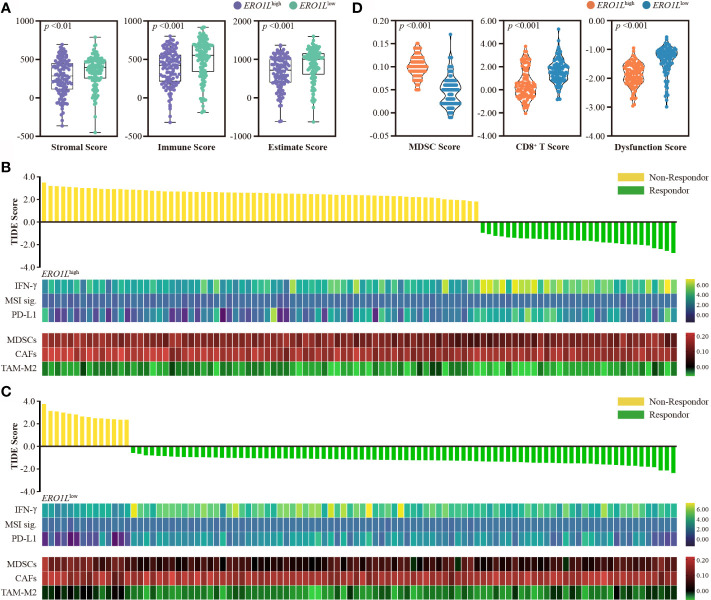
Overexpression of *ERO1L* predicts resistance to immunotherapy. **(A)** Boxplot showing stromal, immune, and Estimate scores within *ERO1L* ^high^ and *ERO1L* ^low^ groups. **(B, C)** Prediction of immunotherapy response using the TIDE computational framework. Other biomarkers for immunotherapy were also assessed. These included: IFN-γ, MSI signatures, PD-L1, MDSCs, CAFs, and TAM-M2. **(D)** Violin plot showing MDSCs, CD8^+^ T, and dysfunction scores within the *ERO1L* ^high^ and *ERO1L* ^low^ groups. Groups are labeled in different colors according to their level of *ERO1L* expression.

To further investigate this hypothesis, the stromal and immune cell infiltration levels were analyzed within *ERO1L* ^low^ and *ERO1L* ^high^ samples using ESTIMATE software. Low expression of *ERO1L* was accompanied with a higher abundance of stromal cells and immune cells in comparison to overexpression of *ERO1L*, which was associated with a significantly higher Estimate score. This suggested that an immune-inflamed TIME that may well be susceptible to immunotherapy (**Figure 5A**). To explore this issue in more detail, the Tumor Immune Dysfunction and Exclusion (TIDE) score was used. This score is a computational framework designed to evaluate the potential of tumor immune escape and is a surrogate biomarker to predict response to immunotherapy. TIDE scores showed that the *ERO1L* ^low^ group had a significantly higher response rate (86.0%) in comparison to the *ERO1L* ^high^ group (31.0%) (**Figures 5B, C**). It was also observed that the *ERO1L* ^high^ group scored high in MDSCs (*p <*0.001) and immune dysfunction (*p <*0.001), while scored low in CD8^+^ T cells (*p <*0.001) in comparison with the *ERO1L* ^low^ group (**Figure 5D**). To validate these results, the MCP counter was applied to quantify the different immune cell populations within the two groups. In agreement with our results, *ERO1L* overexpression scored low in NK cells (*p <*0.001), myeloid dendritic cells (*p <*0.001), neutrophils (*p <*0.001), and endothelial cells (*p <*0.001). *ERO1L* overexpression scored high in B lineage (*p* =0.004), monocyte lineage (*p* =0.020), and fibroblast (*p <*0.001; **Figure S3B**). These results suggest that *ERO1L* is in fact a biomarker with potential applications in the prediction of immunotherapy response in patients with LUAD.

### Mechanisms Underpinning an *ERO1L*-Induced Immune-Suppressive Tumor Microenvironment

**Figure 6:**
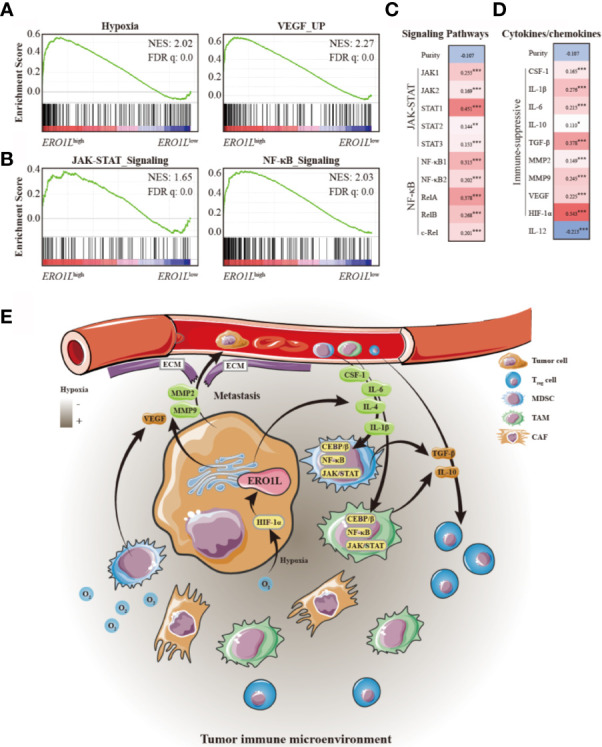
Mechanisms underlying an *ERO1L*-associated immune-suppressive tumor microenvironment. **(A)** GSEA of *ERO1L* ^high^ expressing profiles using a hypoxia signature (NES =2.02, q =0.0) and a VEGF upregulated signature (NES =2.27, q =0.0). **(B)** GSEA of *ERO1L* ^high^ expressing profiles using an upregulated JAK-STAT signature (NES =1.65, q =0.0) and an upregulated NF-κB signature (NES =2.03, q =0.0). **(C)** Confirmation of gene expression changes in the JAK-STAT and NF-κB signaling pathways. **(D)** Correlations between *ERO1L* overexpression, cytokines, and chemokines patterns in the tumor immune microenvironment. **(E)** Schematic showing the *ERO1L*-induced immune-suppressive tumor immune microenvironment. *p < 0.05; **p < 0.01; ***p < 0.001.

Gene set enrichment analysis (GSEA) was performed in order determine whether the transcriptional signature produced by *ERO1L* overexpression was significantly related to other previously studied conditions. By using hallmark gene sets and all curated gene sets as references, GSEA was performed between the *ERO1L* ^low^ group and *ERO1L* ^high^ group in patients from the TCGA cohort. The global expression changes produced in LUAD patients were positively correlated with the signatures of hypoxia (NES =2.02; FDR q-value =0.0) and VEGF (NES =2.27; FDR q-value =0.0; **Figure 6A**). Moreover, GSEA also revealed that the gene signatures of the JAK-STAT (NES =1.65, FDR q-value =0.0) and NF-κB (NES =2.03, FDR q-value =0.0; **Figure 6B**) signaling pathways were commonly enriched when *ERO1L* signaling was upregulated. The expression levels of the components involved in the two pathways were examined, including JAK1, JAK2, STAT1, STAT2, STAT3, NF-κB1, NF-κB2, RelA, RelB, and c-Rel. Consistent with previous findings, there were significant correlations observed between *ERO1L* overexpression and the aforementioned components (**Figure 6C**).

As the JAK-STAT and NF-κB pathways have previously been reported to play a role in increasing the secretion of immune-suppressive factors, we further explored whether *ERO1L* expression could affect the pattern of cytokines and chemokines secreted by tumor cells and infiltrating immune cells, which play a role in shaping TIME. Via TCGA expression profiling analysis, it was identified that cytokines and chemokines secreted by tumors (such as CSF-1, IL-1β, and IL-6), which have been reported to recruit immune-suppressive cells including MDSCs, TAMs, and CAFs, were positively correlated with overexpression of *ERO1L* (**Figure 6D**). Moreover, it was also identified that immune-suppressive cytokines and chemokines (including IL-10, TGF-β, MMP2, MMP9, and VEGF), which are known to be mostly secreted by immune-suppressive cells, were simultaneously in a positive correlation with overexpression of *ERO1L*. Taken together, these data suggest a potential mechanism for *ERO1L*-associated immune-suppressive TIME (**Figure 6E**).

## Discussion

Here, we report a study depicting the biological landscape of *ERO1L* in LUAD. *ERO1L* expression is significantly higher in lung adenocarcinomas in comparison to adjacent normal tissues and is closely related to the prognoses of patients with LUAD. High expression levels of *ERO1L* are associated with a poor prognosis of patients with LUAD. Previous studies have reported that the overexpression of *ERO1L* promoted proliferation, migration, and invasion in pancreatic cancer as well as breast cancer by activating the Wnt/catenin pathway. In this study, overexpression of *ERO1L* was closely associated with infiltrating of immune-suppressive cells and deficiencies in immune-active cells. Therefore, we propose that *ERO1L* functions as an oncogenic factor by inducing an immune-suppressive TIME.

Although *ERO1L* is relatively poorly studied in immunology, molecular studies have investigated the biological functions of the ERO1L protein. This protein is an oxidase in the endoplasmic reticulum which regulates hypoxia-induced oxidative protein folding. Its expression can be induced by hypoxia, which is a common feature of cancers contributing to tumor metastasis, angiogenesis, expansion of tumor-initiating cell, chemoresistance, and genomic instability *via* the regulation of hypoxia-inducible factors such as HIF-1α and HIF-2α. Taking together, these results indicate that *ERO1L* may potentially regulate tumor progression through HIF signaling pathways. In this study, we found that the co-expression module of *ERO1L* took part in oxidation-reduction, glycolytic, and hypoxia. This finding is consistent with previous data. Moreover, hypoxia has also been shown to be an important barrier to effective cancer treatment. We propose that overexpression of *ERO1L* is indicative of a hypoxic TIME, which could potentially confer poor prognoses in patients with LUAD.

*ERO1L* overexpression is closely associated with the infiltration of immune-suppressive cells including MDSCs, TAMs, and CAFs. This leads to an immunosuppressive TIME. MDSCs are derived from bone marrow and have an inhibitory effect on the immune system. They play an important role in tumor immunosuppression, tumor angiogenesis, drug resistance, and tumor metastasis (24). What’s more, MDSCs can produce NO and ROS which can nitrate chemokines and block entry of CD8^+^ T cells to tumors (25). MDSCs have been reported to produce immune-suppressive cytokines including IL-10 and TGF-β, which induce T_regs_ and affecting NK cells (26, 27). Furthermore, MDSCs could eliminate the key nutrition factors needed for T cell proliferation *via* the depletion of L-arginine (28), sequestering L-cysteine (29), or reducing local tryptophan levels due to the activity of indoleamine 2,3 dioxygenase (30). What’s more, recent studies have demonstrated that MDSCs were highly significantly associated with poor OS and PFS in gastrointestinal cancer, hepatocellular carcinoma, lung cancer, and multiple myeloma (31).

TAMs generally display as M2 phenotype macrophages which are devoid of cytotoxic activity, produce growth factors for cancer cells, and have immune-suppressive activity (32). TAMs preferentially localize in the hypoxic areas of tumors, where they promote the expression of the transcription factor HIF-1α. This transcription factor induces the transcription of various elements including VEGF, basic fibroblast growth factor, platelet-derived growth factor, and prostaglandin E2, which is associated with angiogenesis (33). TAMs have potential to produce enzymes and proteases such as MMPs including MMP2 and MMP9 which regulate the degradation of the extracellular matrix (ECM). ECM disruption by TAMs facilitates tumor cell spreading and metastasis (34). What’s more, TAMs also contribute to immune-suppression in the TIME *via* inhibition of IL-12. On the contrary, TAMs promote the secretion of IL-10 and TGF-β, which block T cell proliferation, suppress cytotoxic T lymphocyte (CTL) responses, and activate T_regs_ (35). Clinical studies have demonstrated a strong association between poor survival and increased macrophage density in thyroid, lung, and hepatocellular cancers (36, 37). Similarly, our research proved that in tumors with high expression levels of *ERO1L* are positively associated with the secretion of cytokines and enzymes such as HIF-1α, MMPs, IL-10, TGF-β, and VEGF.

Recently, immune checkpoint inhibitors have led to a paradigm shift in treatment for patients with non-small cell lung cancer (NSCLC). However, the efficacy of these treatments is less than 50%. The clinical responses of ICI are reported to be unfavorable because of the low tumor mutation burden, low PD-L1 expression, and the noninflamed TIME. Based on the results presented in this study, we hypothesized that activation of *ERO1L* signaling could recruit immune-suppressive cells and shape an immune-suppressive TIME and thus conferring resistance to ICI treatment. We propose that *ERO1L* overexpression is an effective biomarker for noninflamed TIME. However, our conclusions were mainly summarized based on the public datasets; further studies based on grafted tumors and patients’ samples are highly needed.

## Conclusion

In summary, our study provides clear insight into the potential role of *ERO1L* in tumor immunology. Our study also suggests the potential prognostic value of *ERO1L* in patients with LUAD. We described that overexpression of *ERO1L*, indicates a hypoxic environment and shapes an immune-suppressive TIME through the recruitment of immune-suppressive cells and inhibition of immune-active cells. High levels of *ERO1L* may be indicative of resistance to immunotherapy. *ERO1L* was shown to associated with cytokine and chemokine patterns in the TIME, which were resulted from activations of JAK-STAT and NF-κB signaling pathways. These findings suggest a potential immune-based anti-tumor strategy *via* the inhibition of *ERO1L* to clear tumor microenvironment infiltrates.

## Data Availability Statement

The datasets presented in this study can be found in online repositories. The names of the repository/repositories and accession number(s) can be found in the article/**Supplementary Material**.

## Author Contributions

JW and HB conceived, designed, and supervised the study. YQ, ZM and XZ collected and visualized the data. LL, CW, and SL performed organoid-related experiments. LL, CW, and PX analyzed the data. LL and CW interpreted findings and wrote the first draft of the manuscript. JW and HB revised and edited the manuscript. All authors approved the final version of the manuscript. JW is the guarantor of this study and accepts full responsibility for the work, had access to the data, and controlled the decision to publish. The corresponding authors attest that all listed authors meet authorship criteria and that no other person meeting the criteria has been omitted. All authors contributed to the article and approved the submitted version.

## Funding

This work was supported by the National Natural Sciences Foundation Key Program [81630071].

## Conflict of Interest

The authors declare that the research was conducted in the absence of any commercial or financial relationships that could be construed as a potential conflict of interest.
