# Supplementary material for: ERO1L Is a Novel and Potential Biomarker in Lung Adenocarcinoma and Shapes the Immune-Suppressive Tumor Microenvironment
Source: Front Immunol. 2021 Jul 20;12:677169. doi: 10.3389/fimmu.2021.677169 (PMC8329662; doi:10.3389/fimmu.2021.677169)
Supplement: Supplementary file 1 [file DataSheet_1.pdf]

ERO1L is a novel and potential biomarker in lung adenocarcinoma and shapes the immune-suppressive tumor microenvironment

Lihui Liu, Chao Wang, Sini Li, Pei Xue, Xue Zhang, Zixiao Ma,
Yiting Sun, Yan Qu, Hua Bai and Jie Wang

Supplementary Material

Table of Content

Title	Content	Page
Table S1	Characteristics of included data sets from GEO database	2
Table S2	Protein expressions of *ERO1L* in HPA database	2
Table S3	Characteristics of included cohorts in PrognoScan database	3
Table S4	Methylation sites mapped to the *ERO1L* gene	3
Figure S1	Expression and methylation profiles of *ERO1L* in LUAD	4
Figure S2	Overexpression of *ERO1L* predicts a poorer prognosis and shapes an immune-suppressive tumor microenvironment	5
Figure S3	Quantifying the different immune cell populations	6

Table S1. Characteristics of included data sets from GEO database

Project	Histology	Stage	Tumor sample	Normal sample	Experiment type	Platforms
GSE7670	LUAD	I-IV	27	27	Affymetrix microarrays	GPL96
GSE31210	LUAD	I-III	226	20	Affymetrix microarrays	GPL570
GSE32863	LUAD	I-IV	58	58	Affymetrix microarrays	PGL6884
GSE19188	LUAD	I-IV	65	45	Affymetrix microarrays	GPL570

Table S2. Protein expressions of *ERO1L* in HPA database

Patient ID	Gender	Age	Histology	Antibody staining	Intensity	Quantity	Location	Antibody used
1847	Male	64	LUAD	Medium	Moderate	>75%	Cytoplasmic/ membranous	CAB034294
1907	Male	73	LUAD	Medium	Moderate	75%-25%	Cytoplasmic/ membranous	HPA026653/ HPA030053
1932	Female	57	LUAD	Medium	Strong	<25%	Cytoplasmic/ membranous	HPA026653/ HPA030053
2003	Female	61	LUAD	High	Strong	75%-25%	Cytoplasmic/ membranous	HPA026653/ HPA030053
2041	Female	51	LUAD	Medium	Moderate	>75%	Cytoplasmic/ membranous	CAB034294
2222	Male	59	LUAD	Medium	Moderate	>75%	Cytoplasmic/ membranous	CAB034294
2403	Female	65	LUAD	Medium	Moderate	>75%	Cytoplasmic/ membranous	CAB034294
3003	Male	49	LUAD	Low	Weak	>75%	Cytoplasmic/ membranous	CAB034294
3391	Female	70	LUAD	Medium	Moderate	>75%	Cytoplasmic/ membranous	CAB034294
4208	Male	75	LUAD	Low	Weak	75%-25%	Cytoplasmic/ membranous	HPA026653/ HPA030053
4923	Male	57	LUAD	High	Strong	75%-25%	Cytoplasmic/ membranous	HPA026653/ HPA030053

Table S3. Characteristics of included cohorts in PrognoScan database

Dataset	Cancer type	Cohort	Endpoint	Patients	Probe ID	HR	95% CIs	*p*
**Jacob-00182-CANDF**	LUAD	CAN/DF	OS	82	218498_s_at	0.94	0.46-1.92	0.87
**Jacob-00182-HLM**	LUAD	HLM	OS	79	218498_s_at	2.31	1.25-4.26	**
**Jacob-00182-MSK**	LUAD	MSK	OS	104	218498_s_at	1.73	0.82-3.66	0.15
**Jacob-00182-UM**	LUAD	UM	OS	178	218498_s_at	1.91	1.36-2.68	***
**GSE13213**	LUAD	Nagoya	OS	117	A_23_P106145	1.30	1.04-1.62	*
**GSE13213**	LUAD	Nagoya	OS	117	A_24_P407311	1.37	1.06-1.78	*
**GSE31210**	LUAD	NCCRI	OS	204	222646_s_at	2.42	1.48-3.98	***
**GSE31210**	LUAD	NCCRI	OS	204	218498_s_at	2.61	1.48-4.58	***
**GSE31210**	LUAD	NCCRI	PFS	204	222646_s_at	2.35	1.63-3.40	***
**GSE31210**	LUAD	NCCRI	PFS	204	218498_s_at	2.70	1.78-4.11	***

LUAD, lung adenocarcinoma; OS, overall survival; PFS, progression-free survival; HR, hazard ratio; 95% CI, 95% confidence interval. * $p<0.05$; ** $p<0.01$; *** $p<0.001$

Table S4. Methylation sites mapped to the *ERO1L* gene

cg02221143	cg04318602	cg05380145	cg05837075	cg08081708
cg08136587	cg08554114	cg10224939	cg10872521	cg13827458
cg15393565	cg18380353	cg18561899	cg21157519	cg23580410
cg26575110	cg05837075	cg08554114		

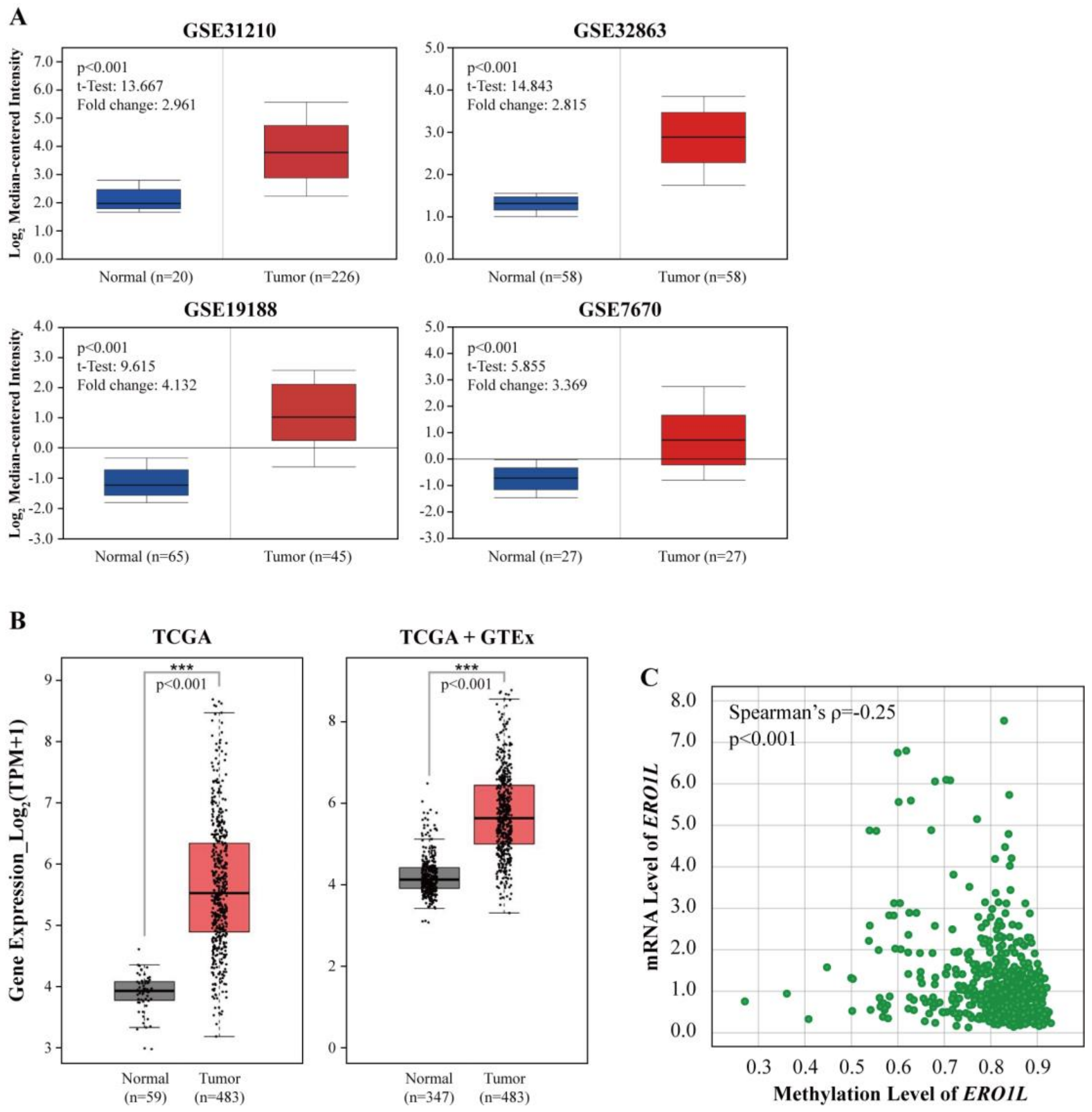

Figure S1. Expression and methylation profiles of *ERO1L* in LUAD. (A, B) Expression levels of ERO1L mRNA in LUAD, compared with normal tissues in the Oncomine database (A) and the TCGA database (B). (C) Correlation analysis between mRNA levels and methylation levels of *ERO1L*. GTEs, Genotype-Tissue Expression. *p <0.05; **p <0.01; ***p <0.001.

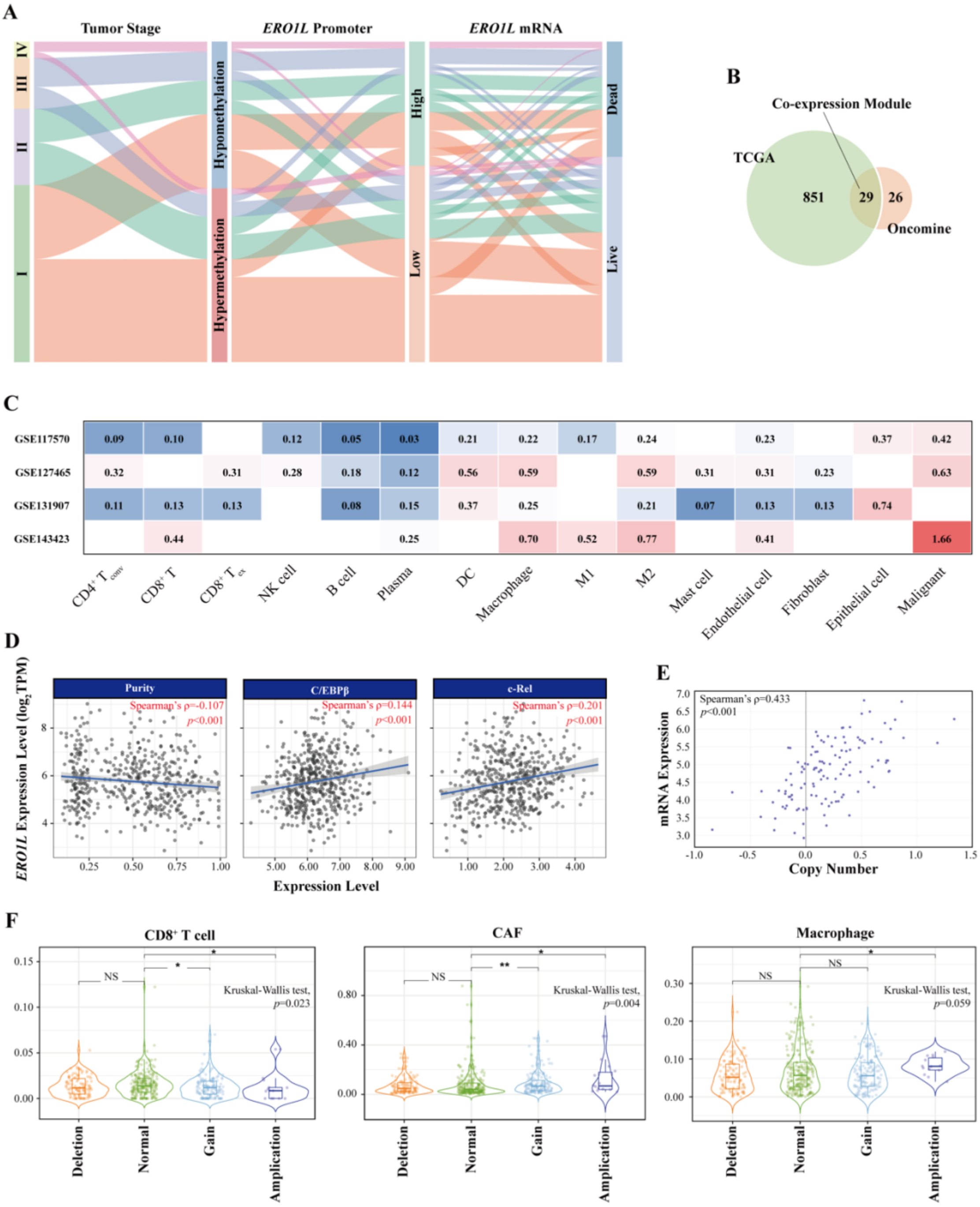

Figure S2. Overexpression of *ERO1L* predicts a poorer prognosis and shapes an immune-suppressive tumor microenvironment. (A) Path analysis described paths and patterns across tumor stages, promoter methylation levels, ERO1L mRNA expression, and survival status. (B) Venn plot for co-expression analysis of expression data from the TCGA program and Oncomine database. (C) Correlation analysis between ERO1L and infiltrating immune cells from four single-cell sequencing datasets. (D) Correlation analysis between ERO1L and C/EBPβ al well as c-Rel. (e,f) High expression level of ERO1L which were resulted from copy number variations (E) and relationships with infiltrating CD8+ T cells, CAF, and macrophages (F). NK, natural killer; DC, dendritic cell; CAF, cancer-associated fibroblast. NS, no significance. *p <0.05; **p <0.01; ***p <0.001.

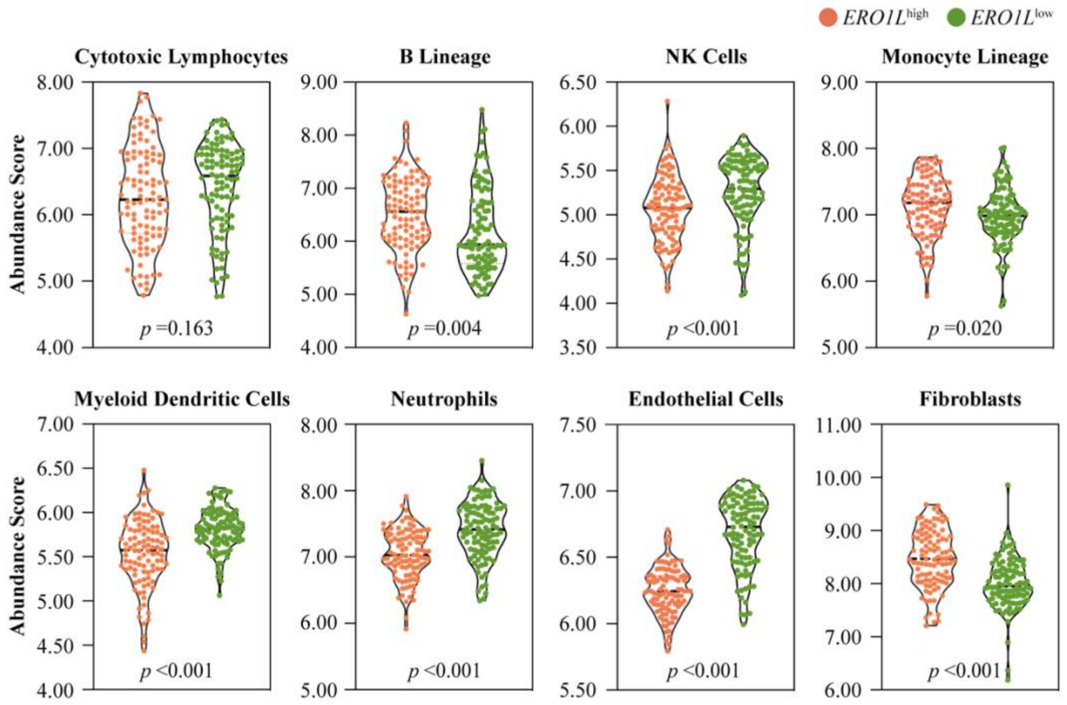

Figure S3. Quantifying the different immune cell populations. Immune cells within the *ERO1L*^{high} and *ERO1L*^{low} groups were calculated using the MCP counter framework. NK cells, natural killer cells.
